# Lymphomatoid Granulomatosis with Isolated Cutaneous Lesions: Prolonged Remission After DA-EPOCH Protocol

**DOI:** 10.4274/tjh.2018.0020

**Published:** 2018-08-05

**Authors:** Vineet Govinda Gupta, Ajay Gogia, Vinod Sharma, Saumyaranjan Mallick

**Affiliations:** 1 Max Super Speciality Hospital, Clinic of Medical Oncology, New Delhi, India; 2 All India Institute of Medical Sciences, Department of Medical Oncology, New Delhi, India

**Keywords:** Lymphomatoid granulomatosis, Remission, Chemotherapy

## To the Editor,

Lymphomatoid granulomatosis (LG) is a rare Epstein-Barr virus (EBV)-related neoplasm that typically presents with pulmonary symptoms and radiographic lung nodules in the majority of patients [1,2]. Frequent skin involvement underscores the importance of a careful cutaneous examination. However, it is rare for a patient to present with isolated skin involvement of this disease. Here we present a case of LG with isolated extensive skin involvement.

A 39-year-old man with no prior known illnesses presented to our oncology clinic located in North India with chief complaints of swellings present over his back over the last 10 years. He had noted the presence of these swellings but had not sought specific therapy as they were essentially asymptomatic except for the cosmetic disfigurement. However, over the last 6 months, there had been a sudden rapid increase in the size of these lesions, associated with ulceration. An associated history of low-grade fever was also reported. No history of significant weight loss or night sweats was described.

**Figure 1:**
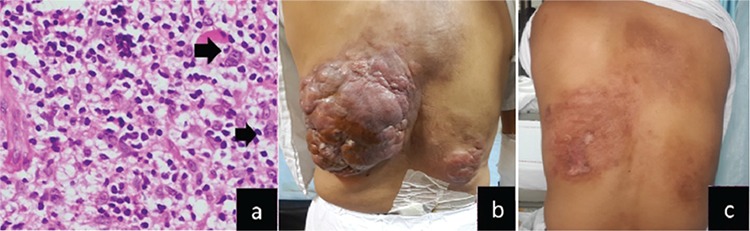
a) Histopathological examination: dermis showing inflammatory infiltrate of lymphocytes and histiocytes along scattered large atypical cells [hematoxylin and eosin, 400^x^ (arrows)]; b) clinical image at baseline presentation; c) clinical image after 4 cycles of chemotherapy.

Upon examination, the patient’s Eastern Cooperative Oncology Group performance status score was 1. A large, hard, lobulated swelling measuring 20 cm in largest diameter with overlying ulceration was present over his left lower back (Figure 1b). The skin surrounding the swelling was indurated. Another swelling with similar morphology measuring 7 cm in largest diameter was noted over the right lower back. A firm, non-tender left axillary lymph node of 2 cm was palpated. The rest of his general and systemic examination was unremarkable.

Based on his presentation, a clinical suspicion of cutaneous lymphoma was entertained and a skin biopsy was performed. On light microscopy, the epidermis was unremarkable. The dermis showed an inflammatory infiltrate of lymphocytes and histiocytes with scattered large atypical cells with areas of necrosis (Figure 1a). Immunohistochemistry demonstrated that the large cells were positive for CD20, CD30, and EBV-latent membrane protein while negative for CD3, ALK1, and EMA. The background population was an admixture of CD3- and CD20-positive lymphocytes. A histological diagnosis of LG was made.

Further staging investigations (contrast-enhanced computed tomography of neck to pelvis, diagnostic lumbar puncture, bone marrow aspiration, and biopsy) did not reveal involvement of any other site. Interestingly, bilateral lung fields were completely normal radiologically. Serum lactate dehydrogenase level was highly elevated at 1516 U/L (normal range: 240-420 U/L). Complete blood counts, liver and kidney function tests, serology for human immunodeficiency and hepatitis B and C viruses, and immunoglobulin levels were within normal limits. Keeping in mind the rapid growth of the disease in a relatively short period of 6 months, a decision was made to treat the patient with six cycles of infusional dose-adjusted etoposide, prednisolone, vincristine, doxorubicin, and cyclophosphamide (DA-EPOCH), drawing from the good experience at the US National Cancer Institute (NCI) with this regimen [1,3,4]. The option of adding rituximab to each cycle was suggested to the patient but could not be done due to affordability issues. Chemotherapy was well tolerated. A clinicoradiological complete remission was documented after 4 cycles (Figure 1c) and reconfirmed after 6 cycles. The patient is now disease-free at 2 years from the end of therapy.

The lung is the predominant site of involvement in LG, with extrapulmonary sites variably involved [1,2]. Skin involvement is seen in 25%-50% of patients and is typically diagnosed concurrently with lung lesions. Isolated skin involvement in the absence of pulmonary disease is unusual, seen in 10%-15% of patients. Skin lesions in LG are variable and include papules, macules, nodules, vesicles, ulcers, alopecia, and ichthyosis [1,5,6]. However, large skin masses as seen in the current case have been seldom described. The unusual presentation in our case could be attributed to neglecting the disease process for a long duration (10 years) on the part of the patient. Occasionally, LG may also transform to an aggressive non-Hodgkin lymphoma, although this was not noted in our histology specimen. There is no consensus on the optimal treatment of LG. Any underlying immunosuppression should be corrected, but no such condition was found in our patient. The treatment of LG is not standardized, and no comparative studies exist for this rare disease. The NCI has demonstrated good outcomes based on DA-EPOCH [1,4] and we decided to follow their protocol with gratifying results.

To conclude, our case demonstrates an unusual isolated cutaneous presentation of LG and is indicative of the clinical heterogeneity of this rare disease. Histopathology remains the key to diagnosis of LG and effective curative therapy is available.

## References

[1] Roschewski M, Wilson WH (2012). Lymphomatoid granulomatosis. Cancer J Sudbury Mass.

[2] Katzenstein AL, Carrington CB, Liebow AA (1979). Lymphomatoid granulomatosis: a clinicopathologic study of 152 cases. Cancer.

[3] Wilson WH, Grossbard ML, Pittaluga S, Cole D, Pearson D, Drbohlav N, Steinberg SM, Little RF, Janik J, Gutierrez M, Raffeld M, Staudt L, Cheson BD, Longo DL, Harris N, Jaffe ES, Chabner BA, Wittes R, Balis F (2002). Dose-adjusted EPOCH chemotherapy for untreated large B-cell lymphomas: a pharmacodynamic approach with high efficacy. Blood.

[4] Shanti RM, Torres-Cabala CA, Jaffe ES, Wilson WH, Brahim JS (2008). Lymphomatoid granulomatosis with involvement of the hard palate: a case report. J Oral Maxillofac Surg Off J Am Assoc Oral Maxillofac Surg.

[5] Rysgaard CD, Stone MS (2015). Lymphomatoid granulomatosis presenting with cutaneous involvement: a case report and review of the literature. J Cutan Pathol.

[6] Tong MM, Cooke B, Barnetson RS (1992). Lymphomatoid granulomatosis. J Am Acad Dermatol.

